# Novel stressors and trait variation determine X-linked meiotic drive frequency

**DOI:** 10.1098/rspb.2025.0426

**Published:** 2025-08-13

**Authors:** Adam M. Fisher, Nicola White, Michael B. Bonsall, Tom AR. Price, Robert J. Knell

**Affiliations:** ^1^ Department of Infection Biology and Microbiomes, University of Liverpool, Liverpool, UK; ^2^ Department of Evolution, Ecology and Behaviour, University of Liverpool, Liverpool, UK; ^3^ Department of Biology, University of Oxford, Oxford, UK; ^4^ School of Natural Sciences, University of Hull, Kingston upon Hull, UK

**Keywords:** sex ratio, meiotic drive, pesticide, suppression, overdominance, heterosis, fitness effects, theoretical models

## Abstract

Sex ratio meiotic drive alleles bias their transmission by impairing the viability of non-drive gametes, leading to skewed population sex ratios. Despite theoretical predictions that drive alleles should reach fixation causing population extinction, meiotic drive persists at intermediate frequencies in wild populations, though the reasons for this are unclear. Here, we investigate how novel environmental stress and genotype-specific fitness costs contribute to drive frequency. Using a suppression-free X-linked meiotic drive system in *Drosophila pseudoobscura*, we exposed flies to varying doses of the pesticide permethrin and measured mortality and fecundity across genotypes. We found that drive-bearing males (SR) and drive-homozygous females (SRSR) exhibited heightened mortality, both in the presence and absence of pesticide, while heterozygous (SRST) females exhibited superior fecundity. Using a mathematical model parametrized with our empirical findings, we explored the long-term population dynamics of meiotic drive under different conditions. Our model predicts that drive frequency has a concave relationship with pesticide dose and is strongly modulated by genotype-specific female fecundity. These results suggest that novel environmental stressors and drive-induced fitness effects play key roles in determining meiotic drive frequencies. Our findings improve our understanding of drive frequencies in the wild and have direct implications for drive-based pest control.

## 1. Introduction

Sex ratio meiotic drive alleles are selfish genetic elements that, when expressed, enhance their intergenerational transmission by diminishing the production and/or viability of non-drive gametes [1]. Meiotic drive can occur in a variety of taxa, both naturally and as the result of synthetic gene editing [2–4]. While female and male gametes (and indeed fungal spores) can be affected by sex ratio meiotic drive alleles, our approach in this study is to focus on male meiotic drive [3]. In males, meiotic drive works by causing the death or dysfunction of non-drive sperm. In heterogametic species, the most studied of which are dipterans, specific drive alleles can be found exclusively on either the X or Y chromosome [2]. As such, in these cases, drive alleles have a transmission advantage over non-drive alleles and can distort the sex ratios of their carrier’s broods with up to 100% efficacy [5]. From here on, ‘meiotic drive’ refers specifically to sex-ratio drivers encoded on the X or Y chromosome that act during male spermatogenesis to bias broods toward one sex.

Meiotic drive can cause a range of evolutionary and ecological consequences on sexually reproducing populations via sex ratio distortion. Early mathematical models of specific drive scenarios showed that, due to their inheritance advantage, drive alleles should reach fixation and cause population extinction due to insufficient mating frequency [6]. Despite these predictions, evidence of extinction in wild populations is inherently hard to verify. In contrast, there is empirical evidence that these selfish genetic elements persist in wild populations at a variety of stable intermediate frequencies [2,3]. Although some progress has been made in understanding why drive persists in natural populations at intermediate frequencies, there remain significant knowledge gaps. A better understanding of the mechanisms regulating drive prevalence in natural populations is crucial for predicting its ecological and evolutionary consequences.

One mechanism that can prevent meiotic drive alleles from reaching fixation is the evolution of drive suppressors. As drive-induced sex ratio distortion becomes increasingly severe, selection for the production of gametes that create the rare sex becomes stronger [7]; this can lead to the evolution of molecular mechanisms that suppress drive. Indeed, several studies using field-collected *Drosophila *spp. have shown intermediate drive prevalence (less than 60%) in populations where drive suppression was detected [8,9]. However, such evidence of a relationship between suppression and intermediate drive frequencies is correlative, and there are many examples of natural drive systems in which there is no evidence of suppression [10,11]. For example, the fruit fly species *Drosophila pseudoobscura* and *Drosophila neotestacea* have been surveyed extensively and appear to maintain stable intermediate drive frequencies, despite an apparent lack of suppression [12,13]. This diminishes the extent to which drive suppressors can be regarded as widespread evolutionary mediators of drive prevalence.

An additional explanation for intermediate drive prevalence is the limitation of drive transmission via drive-associated fitness costs. The most studied of these costs is reduced sperm count per ejaculate in drive males due to the killing of non-drive gametes. Reduced sperm count has been demonstrated in several species and can lead to lower paternity in drive relative to non-drive males [14–16], reducing drive prevalence. While this can explain the prevention of drive fixation in polyandrous species, where males experience sperm competition with one another, it cannot explain the intermediate prevalence of drive seen in some monandrous species [17]. In addition to competitive costs, drive alleles may reduce carrier fitness via their tendency to accumulate deleterious mutations [18]; however, there has been little attempt to directly measure fitness in response to the accumulation of such mutations. To explain why drive persists at intermediate frequencies across many species, we must identify drive-associated traits that are universally influential for organism fitness. While there have been some studies investigating the impact of drive on such traits (e.g. fecundity), these are scarce, and their conclusions remain mixed [19–22].

In addition to identifying the impact of drive on fundamental fitness traits, our ability to explain the persistence of drive could be further expanded by understanding how the frequency of drive is affected by ecological factors and/or environmental change. Human activity is increasing the rate at which wild populations encounter novel environmental stressors, and this can lead to large shifts in the genetic landscape of a population [23,24]. If meiotic drive negatively impacts fitness at the level of the organism, then novel stressors may exacerbate these negative fitness consequences for drive frequency. Establishing a link between drive and a reduced resistance to novel environmental stress could not only provide a widely applicable explanation for a lack of drive fixation, it could also help explain variance in drive frequency across wild populations. Several studies have measured how variation in certain ecological parameters, such as temperature and population density, impacts drive frequency [25,26]. However, to the best of our knowledge, there are no studies that measure the impact of acute novel stress on meiotic drive frequency.

In this study, we exposed drive and non-drive carrying fruit flies to a novel environmental stressor, the pesticide permethrin, at varying intensities. We measured how the presence of the drive allele interacted with the impact of the stressor on two life-history traits that are fundamental for determining fitness: mortality rate (survival) and fecundity (eggs per female). We then extrapolated these empirical findings to parametrize a simple mathematical model; this allowed us the explore the interactive effects of meiotic drive and novel environmental stress on drive frequency at the population level.

## 2. Methods

### (a) Empirical methods

#### (i) Stock population maintenance

We used an X-linked meiotic drive in *D. pseudoobscura* as our model system. SR is used to denote the presence of the meiotic drive allele, and ST is used to denote the presence of a non-drive or ‘wild-type’ allele at the same locus. Thus, there are five possible genotypes: (i) SR—males carrying the drive allele, (ii) ST—‘wild-type’ males, (iii) SRSR—females homozygous for the drive allele, (iv) STST—females homozygous for the wild-type allele and (v) SRST—heterozygous females. Note that males are denoted by a single allele due to them only having one X-chromosome. *Drosophila pseudoobscura* was chosen due to ease of husbandry and the fact that they are not known to evolve mechanisms for suppressing gene drive. The *D. pseudoobscura* stocks used in this experiment were descendants of 120 females collected from a natural population in Arizona, USA in 2004 [16] and have been maintained as a large outbred population since. In this experiment, we continued to limit inbreeding by keeping large experimental populations (*n* > 400) in appropriately sized (250 ml) glass bottles rather than vials. To ensure the persistence of all genetic classes in our populations, breeding was organized by genotype. Specifically, several hundred SRSR females and SR males from our outbred population were crossed to produce SRSR females (typically, males constitute less than 5% of these broods owing to the X-linked drive), SRSR females were crossed with ST males to produce SR males and SRST females, and STST females were crossed with ST males produced STST females and ST males. Random individuals from each genotype were selected for polymerase chain reaction to confirm their presumed genotypes (methods and primers reported in [27]).

#### (ii) Exposure to stressor

We chose to use pesticide as our novel environmental stressor for several reasons. First, unlike stressors such as high temperatures or drought, which can be introduced accidentally in laboratory environments, we were certain that pesticide was indeed a novel stressor. In addition, pesticides are an ever-growing threat to wild arthropod populations; hence, our results are directly relevant to a wide range of ‘real-world’ scenarios [28,29]. Finally, gene drives are frequently cited in relation to their ability to act as agents of pest control [30]. Using gene drives for this purpose may mean that they are used on populations that are subject to additional control methods such as pesticides. Thus, understanding how pesticide exposure impacts the frequency of gene drive is important for predicting the scenarios in which gene drive will be most effective for suppressing pests.

Our pesticide of choice was permethrin owing to its low toxicity to humans and common usage for the suppression of real-world pest populations. We made up a stock solution by dissolving permethrin 25 : 75 trans–cis mixed isomer (Scientific Laboratory Supplies Ltd.) in acetone to a concentration of 11.5 μg ml^−1^. We then evenly applied a set quantity of the acetone-permethrin solution to the inside of 27 × 72 mm glass fly vials. The vials were left for 15 min to allow the acetone to evaporate, leaving behind the permethrin precipitate. The amount of the permethrin-acetone solution used varied between 0−1.8 ml across vials; this was done to achieve variation in the permethrin concentration per vial. Control vials were coated with 1 ml pure acetone only. Our concentration range was informed by pilot trials on *D. pseudoobscura* and was selected to capture a range of exposures in which mortality varied from 0 to 100%. A set number of flies (the number varied between 7 and 11 depending on availability) were anaesthetized using CO_2_ and allocated to the permethrin-treated vials according to genotype. They were then left in the permethrin vials for 1 h before being returned to the original vials from which they came. After 72 h, we recorded the number of survivors (all of which had been treated with permethrin) in each vial. To control for the potential confounding effect of age, all individuals had emerged as adults 7 days prior to exposure. In addition, males and females were separated as soon as possible post-emergence to maximize the likelihood that all experimental individuals were virgins. The total sample size (number of vials) for these exposures was N=175, with n=35 for each genotype.

#### (iii) Measuring female fecundity

Females from all three genotypes were challenged with pesticide using the method and concentration range described above. Surviving virgin females were then moved to a new vial and paired with two non-exposed ST males for 7 days to mate, after which the two males and the female were removed from said vial. After 30 days, the number of emerged adults was counted in each vial. As we did not monitor the females for the entirety of their time with the male, we cannot be sure they mated; as such, vials with zero newly emerging adults were discounted as they do not necessarily indicate zero fecundity. Moreover, as measuring entire lifetime fecundity is time-consuming (this would involve keeping all females until they died and counting all of the offspring produced), we opted to take two fecundity measurements per female; this was done with the intention of producing a reliable proxy for fecundity. We understand that this approach ignores potential cumulative fecundity effects of genotype and pesticide acting over the entire adult female lifespan. However, this simply means that our findings are likely to be conservative estimates of the true impacts of genotype and pesticide. To measure fecundity, females were transferred to a second vial immediately after their time in the first vial and, as before, were kept there for a week with two ST males. As previously, females were removed from the second vial after a week, and any emergent adults were counted 30 days later. Sample sizes (female replicates) were $n_{SRSR}=146,\;n_{STST}=238$ and $n_{SRST}=368$ (note that every effort was made to distribute our samples as evenly as possible over the three genotypes; however, due to genotype-specific survival differences, it was impossible to maintain consistency in the number of surviving females from which we could measure fecundity).

#### (iv) Statistical analyses

Mortality data were analysed using a generalized linear model with binomial errors. Relative survivor and death counts (per vial) formed the response variable, and sex, genotype, permethrin dose, and the dose by genotype interaction were the fixed effects in our maximal model. Model selection was conducted using likelihood ratio tests, and non-significant terms were sequentially dropped from the model to generate a minimum adequate model.

Fecundity data were analysed using a generalized linear mixed effects model (GLMM) with a Poisson error distribution. The maximal model used offspring count as the response variable, with permethrin concentration, genotype and their respective interaction as the fixed effects. As measurements were taken from multiple individuals from the same vials, and two measurements were taken per individual, we included a random intercept effect in which individual was nested within vial. Model selection was again conducted using likelihood ratio tests in which non-significant terms were sequentially dropped from the model. A post hoc test was conducted using the emmeans package in R [31] to examine pairwise comparisons of genotype effects. Estimated marginal means were calculated on the logit scale (from our GLMM), and pairwise contrasts were tested using *z*-tests with Tukey adjustment for multiple comparisons.

For all empirical analyses, model fit and the presence of overdispersion were checked by graphically analysing quantile–quantile plots of residuals and plots of residuals versus observation index (see electronic supplementary material). All analyses were run in R v. 4.2.3 [32], and mixed effects models were generated using the lme4 package [33].

### (b) Theoretical methods

#### (i) The model

To explore the population-level impacts of our empirical findings over large timescales, we derived a class-structured deterministic mathematical model that tracks the abundance of genotypes across generations. Our model is deliberately simplistic to allow for a clear delineation of cause-and-effect between the fitness traits quantified in our empirical experiments and SR dynamics. The genotypes and inheritance rules mirror those of our empirical study system, and the efficacy of the drive mechanism is assumed to be 100%. We assume non-overlapping generations; thus, beyond our experimental system, our model is most relevant to seasonal pest populations in temperate regions. For simplicity, the model assumes that females only mate once and produce one brood per generation. Females always mate as long as the male population is greater than zero; furthermore, mating occurs randomly, and as such, the frequency of genotype pairings is directly proportional to their relative abundance. Each generation, individuals are exposed to pesticide before mating, this results in a proportion of each genotype dying prior to reproducing. The probability of death depends on the individual’s genotype and the dose of pesticide in the environment, such that $d_{i,j}$ is the probability of death for genotype j when they encounter pesticide at dose i. When present, pesticide is assumed to be spread homogeneously across the environment; as such, each genotype encounters the same dose for any given time point. The number of new individuals produced per mating is determined by the genotype of the female such that $b_{j}$ is the number of offspring produced per brood by a female of genotype j. The model is density dependent, and the strength of density dependence is determined by a single parameter (θ). A detailed derivation of the model can be found in the electronic supplementary material.

#### (ii) Parametrization and analysis

The purpose of our model is to extrapolate our empirical findings to the population level and observe how these mechanisms influence the frequency of SR males in the population. We parametrized our model directly in line with the results of our laboratory experiments. Specifically, the predicted means generated from our empirical analysis (see above) were used directly to create mortality reaction norms that parametrized the death rate in our mathematical model. In other words, the value of parameter $d_{i,j}$ in our simulations was the exact mortality rate predicted by our empirical analysis for genotype j when exposed to dose i. Values for dose i followed the range used in the empirical experiments. Similarly, parameter values for female fecundity were adopted directly from our empirical findings to inform female fecundity by genotype in our mathematical model. Here, the mean fecundity for a given genotype across all exposure concentrations, as determined by the empirical work, was used as the median for the parameter range in our mathematical model. The fecundity ranges in our mathematical analysis were set to the median fecundity ±10 offspring to simulate natural variation.

To analyse our model, we used simulations to observe how the equilibrium frequency of SR males $\left(\frac{SR}{SR\;+\;ST}\right)$ varied in response to pesticide dose and female fecundity. In our model, all parameters are positive and $d_{i,j}$ never exceeds 1; moreover, the model has a built-in saturating mechanism in the form of density-dependent population growth (see electronic supplementary material). As such, the model is inherently asymptotic. We can, therefore, assume the model will reach an equilibrium given enough time. Also, based on empirical evidence from other dipteran species in which drive reaches equilibrium in less than 20 generations [34,35], we assume that the SR frequency at t=1000 approximates the equilibrium frequency. We used this metric to simulate how pesticide dose and genotype-specific female fecundity interact to determine the equilibrium frequency of all SR males and SR-homozygous females. All mathematical analyses were performed using Jupyter Notebooks [36] running on a Python 3 kernel.

## 3. Results

### (a) Empirical findings

#### (i) Pesticide impact on mortality

**Figure 1.**
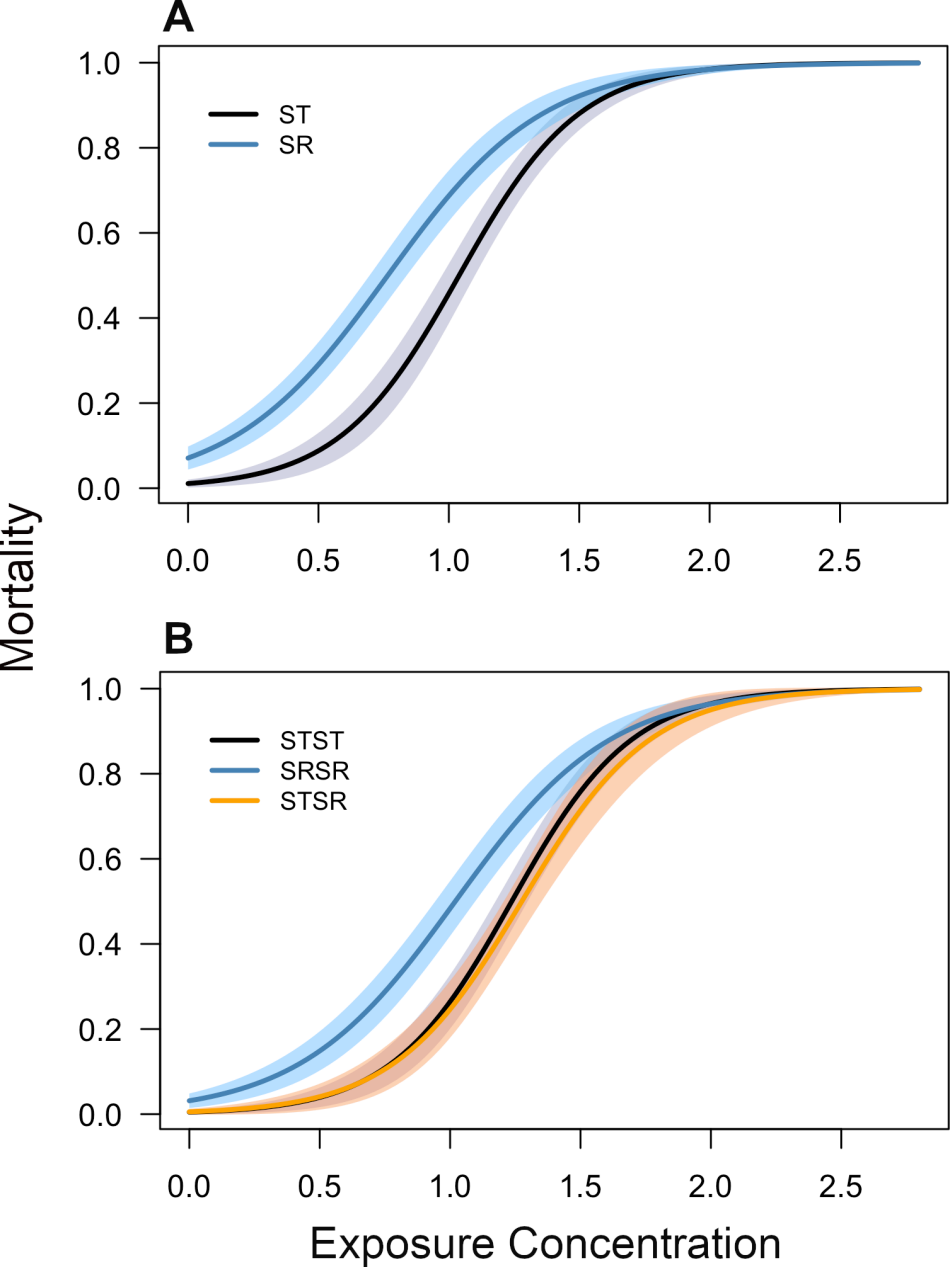
Mortality in response to permethrin exposure (measured as ml of 11.5 μg ml^−1^ solution) in male (A) and female (B) *D. pseudoobscura*. Line colours indicate the genotypes of individuals based on the presence of the X-chromosome driver (SR), and in females specifically, to separate SR and wild-type (ST) homozygotes from heterozygotes. Lines represent predicted means from our statistical model, 95% confidence intervals shown as shadings around each line.

For our analysis of the mortality data, the best fitting model was our maximal model containing fixed effects terms for permethrin dose, sex, genotype and the dose by genotype interaction. SR males and SRSR females consistently had higher mortality than other genotypes of the same sex (figure 1). In addition, the interaction between dose and genotype was significant (d.f. = 2, $\chi^{2}$ = 6.53, *p* = 0.038), highlighting the presence of genotype-specific mortality responses. Unsurprisingly, the main effect of permethrin dose was highly significant (d.f. = 1, $\chi^{2}$ = 335.11, *p* < 0.0001), as was the main effect of genotype, highlighting significant genotypic differences at the model intercept (d.f. = 2, $\chi^{2}$ = 22.31, *p* < 0.0001). Sex significantly affected mortality, with males being more sensitive to permethrin than females (d.f. = 1, $\chi^{2}$ = 26.41, *p* < 0.0001).

#### (ii) Pesticide impact on female fecundity

**Figure 2.**
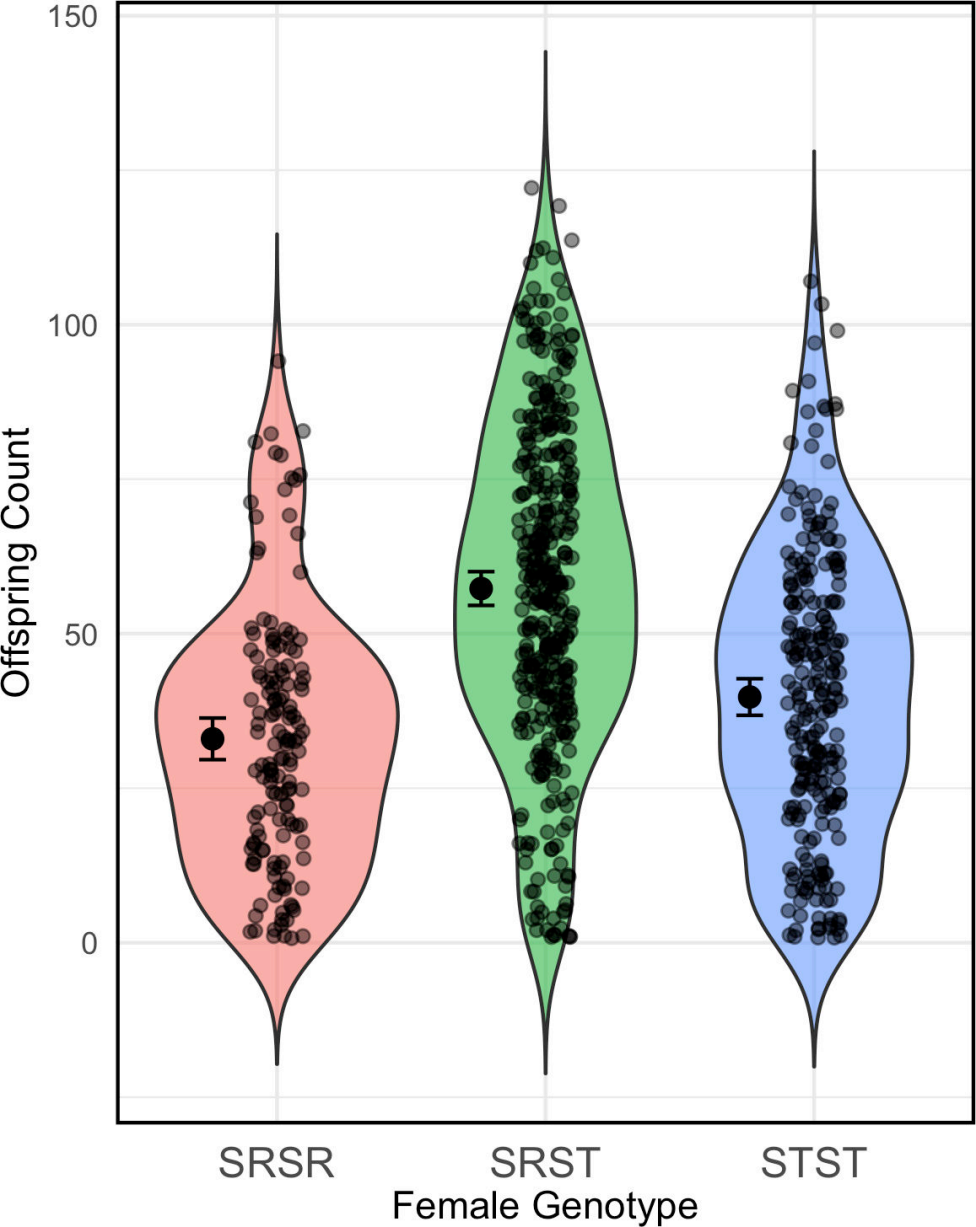
Fecundity (measured as offspring count after a week of egg laying) varies across females depending on genotype in *D. pseudoobscura*. Means and 95% confidence intervals are shown to the left of the raw data for each genotype.

Upon analysing our fecundity data, the maximal model had a singular fit due to negligibly small variance in the nested random effect ‘individual’. Thus, we simplified our random effect structure to include a random intercept of ‘vial’ only. The interaction between exposure concentration and genotype was non-significant (d.f. = 2, $\chi^{2}$ = 0.055, *p* = 0.973), meaning the relative fecundity of each phenotype did not vary significantly with exposure concentration. Therefore, the interaction was dropped from the model, leaving us with our final model that included significant terms for both exposure concentration (d.f. = 1, $\chi^{2}$ = 13.23, *p* = 0.00028) and genotype (figure 2; d.f. = 2, $\chi^{2}$ = 43.29, *p* < 0.0001). Our post hoc test revealed that all pairwise comparisons of genotype-specific fecundity were significant. Specifically, SRST females were more fecund than SR homozygotes (estimate = 0.63, s.e. = 0.086, *z* = 7.27, *p* < 0.0001) and ST homozygotes (estimate = 0.384, s.e. = 0.074, *z* = 5.193, *p* < 0.0001) and SR homozygotes were less fecund than ST homozygotes (estimate = −0.243, s.e. = 0.089, *z* = −2.74, *p* = 0.017).

As noted in the methods, the relative proportions of each genotype were not consistent across the exposure concentration range. Moreover, we were unable to get fecundity data for SRSR females at the two highest exposure concentrations owing to reduced survival (figure 1B). Thus, despite not finding a significant interaction between genotype and exposure concentration, the visual representation of the overall impact of exposure concentration on fecundity is potentially misleading due to the confounding effect of genotypic differences in survival. As the relative sample size of each genotype varies with dose, any visual representation of an ‘overall’ trend between exposure concentration and fecundity must be treated with caution. Therefore, this figure is presented only in the electronic supplementary material of the empirical analysis. Similarly, the confounding effect of survival is also likely to bias the genotype-specific fecundity means taken across all exposure concentrations (figure 2). However, in this case, the bias simply means that our findings are conservative, as the observed fecundity benefit of heterozygosity would probably have been even larger if SRSR and STST fecundity were measured to a greater extent at high pesticide concentrations. Similarly, given that the STST sample size was larger than the SRSR sample size at high concentrations, our result showing greater fecundity in STST relative to SRSR females (figure 2) is also likely to be an underestimate (electronic supplementary material).

### (b) Theoretical findings

**Figure 3.**
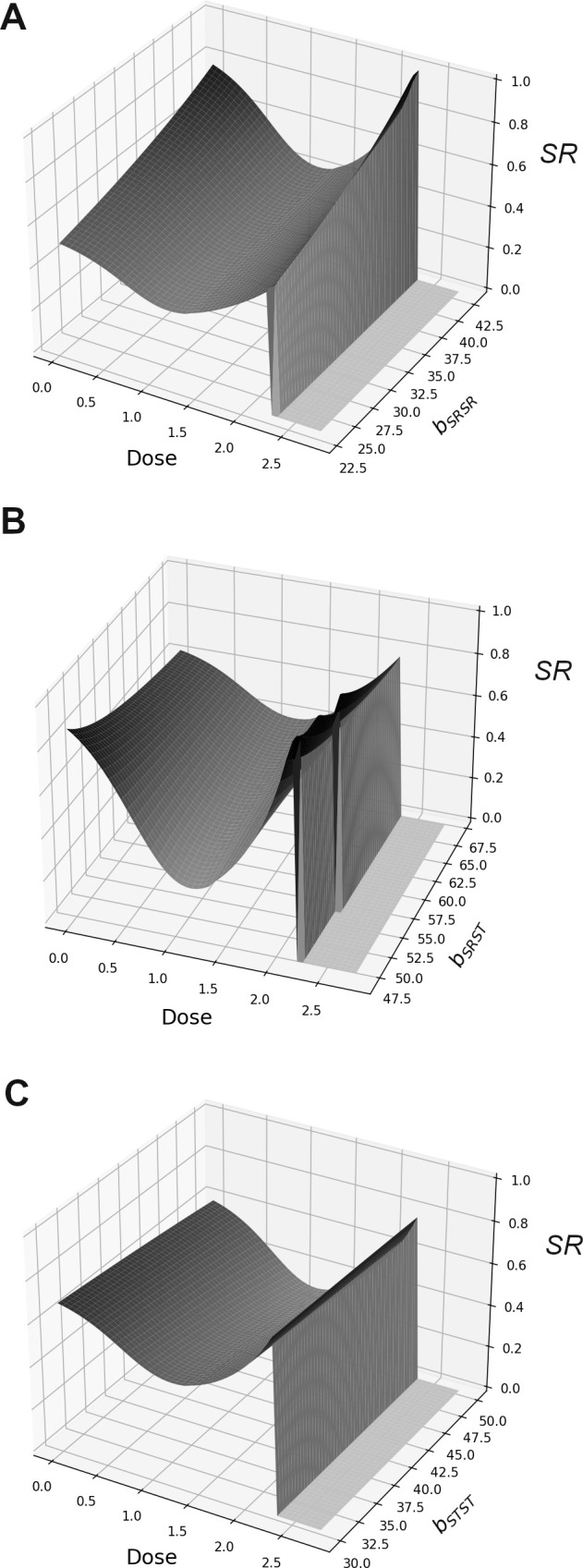
Variation in equilibrium SR frequency (the proportion of the male population carrying the SR drive allele) in response to pesticide dose (measured as ml of 11.5 μg ml^−1^ solution) and genotype-specific female fecundity (b). Panel (A), displays results for variation in the fecundity of SR-homozygous females ($b_{SRSR}$), panel (B) displays results for fecundity variation in heterozygous females ($b_{SRST}$) and panel (C) displays results for fecundity in ST-homozygous females ($b_{STST}$). Default fecundity values (when not being varied) were the mean value for the corresponding genotype-specific fecundity recorded during our laboratory experiments (figure 2).

Our model predicted that male SR persists in a population up until the pesticide dose is lethal for all genotypes (greater than approx. 2.3, figures 1 and 3) and causes extinction. Moreover, the extinction threshold was not affected by male SR prevalence, as shown by the fact that extinction is achieved at the same point across all simulations (figure 3). The model also predicted that male SR never reaches fixation and the population never went extinct due to lack of males; this is probably due to fixation being prevented by higher mortality (compared with other genotypes) in male SR and female SRSR genotypes, even when pesticide dose was zero (figure 1).

Overall, male SR frequency had a concave relationship with pesticide dose, this was true across all values of female fecundity (figure 3). Variation in the fecundity of heterozygous females and SR-homozygous females ($b_{SRST}$ and $b_{SRSR}$, respectively) strongly interacted with pesticide dose to modulate male SR frequency. Specifically, lower values of $b_{SRSR}$ corresponded to a persistent reduction in male SR frequency across all pesticide doses compared with higher values of $b_{SRSR}$ (figure 3A). Variation in $b_{SRST}$ did not have a strong effect on male SR frequency at the extremes of the pesticide dose range, but low values of $b_{SRST}$ did reduce male SR frequency considerably at intermediate pesticide doses (figure 3B). Finally, variation in $b_{STST}$ did not have a considerable impact on the relationship between pesticide dose and male SR frequency (figure 3C).

**Figure 4.**
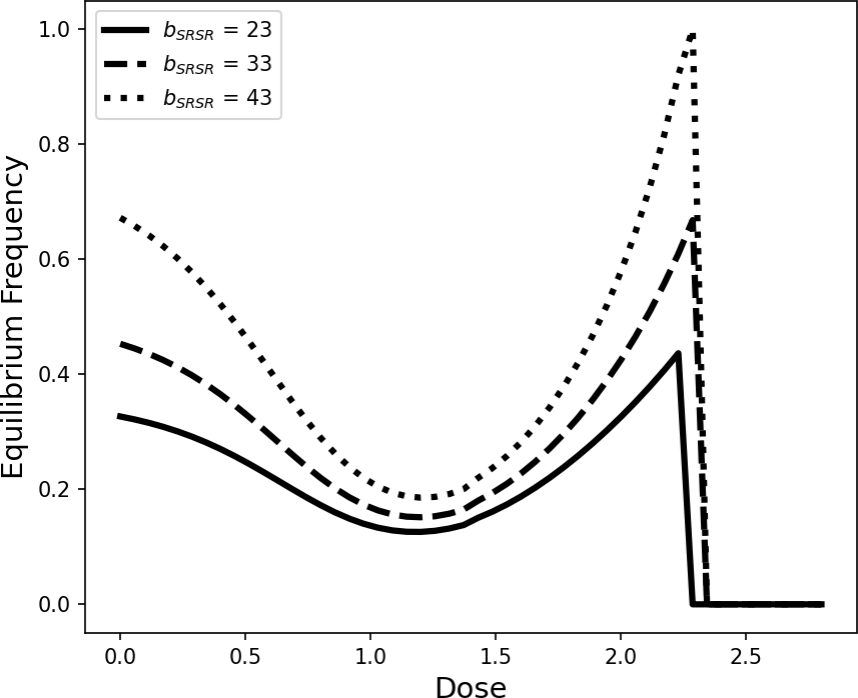
Variation in equilibrium frequency of SRSR females in response to pesticide dose (measured as ml of 11.5 μg ml^−1^ solution) and SRSR female fecundity ($b_{SRSR}$). Default fecundity values for other genotypes were the mean value for the corresponding genotype-specific fecundity recorded during our laboratory experiments (figure 2).

Similarly, the equilibrium frequency of SRSR females had a concave relationship with pesticide dose, and the shape of this relationship was modulated by variation in female fecundity, though the overall trend did not vary (figure 4). Specifically, the equilibrium frequency for SRSR females was consistently higher across the entire pesticide dose range as SRSR fecundity ($b_{SRSR}$) was increased.

## 4. Discussion

Our understanding of why meiotic drive persists in the wild at intermediate frequencies and does not always reach fixation remains incomplete. This is particularly true for populations where drive suppression mechanisms have not evolved. In addition, where drive is present, we are unable to explain the observed temporal and spatial variations in frequency. Here we show that, in a system where any meiotic drive suppression mechanism is absent (though see the prediction of Price *et al*. [16] that polyandry itself may represent a drive suppression mechanism), drive frequency can be modulated through interactive fitness consequences of the drive (SR) allele at the individual level. Specifically, we show that SR males and SR-homozygous females have higher mortality rates relative to other genotypes and that these mortality differences change in response to increasing levels of novel environmental stress (pesticide, figure 1). We also show that SR-homozygous females exhibit reduced fecundity. However, perhaps surprisingly, heterozygous females did not display higher mortality relative to ‘wild-type’ (ST-homozygous) females and were considerably more fecund than both SR homozygotes and ‘wild-type’ females (figure 2); this suggests the fitness costs associated with SR are fully recessive in females, and that a single copy of the drive allele can induce a considerable fecundity benefit. Using our mathematical model, we show that these individual-level fitness impacts have strong isolated and interactive effects on the frequency of SR at the population level (figure 3). These findings improve our mechanistic understanding of drive dynamics in the wild and are relevant for assessing the impact of novel environmental stressors on drive frequency. Our results are also directly relevant for predicting drive frequency in populations treated with pesticide.

### (a) SR can increase mortality

In the current study, significant differences in our model intercepts (figure 1) show that the presence of the SR allele, even in the absence of pesticide, has a deleterious effect on survival in both males and females; though in females, this effect only occurred in SR homozygotes (figure 1B). It is well documented that multiple loci can be involved in facilitating the action of X-linked meiotic drive. As a consequence, there is strong selection for drive chromosomes to preserve the mechanism of action by increasing their inversion rate in order to limit recombination; this leads to non-random association between alleles (linkage disequilibrium). A reduction in recombination inevitably lowers the intergenerational rate of genetic change and may lead to maladaptation due to a diminished evolutionary rate and the accumulation of deleterious alleles [1,18]. We did not quantify the presence of deleterious alleles in this study, but it is very possible that the reduction in survival seen in SR individuals in this study is due to drive-induced linkage disequilibrium.

Although there is good reason to believe that SR alleles are inherently detrimental to the fitness of their carriers, females in this study only suffered a reduction in survival when they were SR-homozygous. Heterozygous and ST-homozygous females did not differ significantly in terms of survival (figure 2B). Our evidence, therefore, suggests that the fitness costs of drive, at least in terms of mortality, are recessive. Indeed, several previous studies corroborate this finding [20,37,38]. For example, drive alleles are known to induce sterility in house mice but only in homozygotes [38]. In addition, SR-homozygosity has been shown to increase mortality in *Drosophila melanogaster* [37] and *D. pseudoobscura* [26].

Unsurprisingly, pesticide dose had a strong positive impact on mortality across all genotypes (figure 1). Perhaps less expected is the nature of the interaction between genotype and pesticide dose. When pesticide dose was zero (controls), mortality was higher in both SR males and SR-homozygous females compared with other genotypes of the same sex. In males, these survival differences appear to converge at higher pesticide doses (figure 1). This means that, although SR individuals have higher baseline mortality than ST individuals, their mortality rate is less sensitive to increasing pesticide dose. Although this result may seem counterintuitive, it is not entirely unexpected. Some theoretical and empirical studies suggest that novel environmental pressures can mitigate the negative fitness effects of previously deleterious alleles [23]. For example, fitness differences between mutant and wild-type bacterial strains are reduced in response to exposure to novel chemical stressors [39]. A similar trend was observed in females, although mortality differences between SRSR and the other genotypes seemed to increase slightly at intermediate pesticide concentrations (figure 1B). This disparity in the sex by genotype mortality response is not straightforward to interpret, and it is impossible to understand the mechanism behind this trend based on our data. However, sex-specific responses to novel stressors have been observed previously, including in *Drosophila *spp. [40–42]. We suspect that fundamental physiological differences in the detoxification responses between males and females are at least partly responsible for our observed trends [42,43]. Similar experiments on other species would be illuminating in this regard.

### (b) SR alleles influence female fecundity

The evidence regarding the impact of meiotic drive alleles on female fecundity is highly mixed. We show that fecundity in SR homozygotes is reduced relative to ST homozygotes; this finding corroborates recent work on *D. pseudoobscura* (by Larner *et al.* [20]). However, unlike Larner *et al.* [20] who found no significant difference in fecundity between SRST and STST genotypes, we found that SRST females were considerably more fecund than both SRSR and STST females (figure 2). This finding also contrasts with Dyer & Hall [44], who observed reduced fecundity in SRST females relative to STST females in *Drosophila recens*. Although our results disagree with recent evidence, two previous studies found similar patterns of fecundity across female genotypes [19,26]. Here, Curtsinger & Feldman [19] compared offspring production across different populations of 120 adult *D. pseudoobscura* (50 : 50 sex ratio) in which female genotype varied between SRST, STST and SRSR. Like the current study, Curtsinger & Feldman [19] found fecundity to be greatest in populations of SRST, followed by STST and then SRSR. Wallace [26] also found heightened fecundity in SRST females relative to SRSR and STST in a laboratory population of *D. pseudoobscura*; however, Wallace [26] provides no formal statistical testing of these fecundity differences.

Larner *et al.* [20] suggest that their results differ from those of Curtsinger & Feldman [19] due to considerable differences in methodology. This is a valid point, given that Curtsinger & Feldman [19] measured population-level fecundity rather than fecundity per female, increasing the probability of measurement error. Similarly, Wallace [26] measured fecundity per vial containing multiple females. However, like Larner *et al.*, our methodology directly measured fecundity per individual across many individuals from each genotype. As such, our results provide a reliable indication that SR drive alleles can provide fecundity benefits to females via heterosis (hybrid vigour).

Although our results cannot confirm the mechanism behind the observed genotype-specific fitness differences, there are several mechanisms that seem plausible. The diminished fecundity seen in SRSR females can be explained by the increased inversion rate observed in X-linked drive alleles. As mentioned earlier, this reduces recombination rate, resulting in the accumulation of deleterious recessive alleles. Moreover, genetic disruption occurring at inversion break points may change the expression of certain fecundity-related genes by repositioning them into novel chromatin environments [45]. Thus, it is possible SRST fecundity is higher than that of SRSR due to the presence of the ST allele which (i) masks the effects of deleterious recessive SR alleles and (ii) reduces the disruption of epigenetic processes created by SR inversion break points. Furthermore, the presence of SR in SRST heterozygotes could be masking the effects of deleterious recessive alleles expressed on the ST chromosome, leading to the observed fecundity disparity between STST and SRST females. Increased fecundity due to heterosis has been demonstrated in many other species, including other *Drosophila *spp. [46,47], but the direct impact of heterosis on population-level SR frequencies has been largely unexplored. In addition, future work could focus on chromosome-wide gene mapping combined with transcriptomics to mechanistically demonstrate how SR impacts fitness, both in terms of deleterious allele accumulation and possible disruption to gene expression.

### (c) Novel stress and female fecundity interact to determine SR dynamics

Results from our mathematical model suggest that SR in this system can never reach fixation (figure 3); this is due to increased mortality, even in the absence of pesticide (figure 1), reducing the fitness of drive individuals relative to non-drive individuals. In addition, as mortality rates between drive and non-drive individuals converge at high pesticide doses, drive did not go extinct prior to entire population extinction (figures 1 and 3). Therefore, our model predicts that the presence of drive at intermediate frequencies is robust to variation in the intensity of this novel stressor.

Given the fact that mortality rates between drive and non-drive males converge at higher pesticide doses (figure 1A), one could reasonably predict that SR frequency would increase consistently with pesticide dose. However, our mathematical model predicts a concave relationship between SR frequency and pesticide dose (figure 3). In X-linked gene drive systems, the frequency of all SR-bearing genotypes are inextricably linked to one another because all SR-bearing genotypes contribute directly to each other’s propagation (electronic supplementary material). Thus, the concave relationship between the SR-male frequency and pesticide dose is a direct consequence of increased mortality in SRSR females relative to other female genotypes at intermediate pesticide doses (figures 2B and 4).

The extent to which SR frequency is reduced at intermediate frequency is affected by variation in SRSR and SRST fecundity, such that when SRST and SRSR fecundity is low, the frequency of drive is consistently lower than when SRSR and SRST fecundity is high (figure 3A,B). This finding is significant in two major ways. First, it demonstrates the potential for interactions between an environmental stressor and genotype-specific trait variation to generate large variation in drive frequency. Second, it directly illustrates the precarity of the impact that pesticide use has on the frequency of drive. The introduction of gene drives has been proposed as a potential method of pest control (e.g. [30]). Our results highlight that, if drive is to be used alongside pesticide treatment as part of combined control strategy, intermediate levels of pesticide should be avoided in order to preserve drive frequency.

### (d) Assumptions and limitations

Our study does not measure (empirically or theoretically) the impact of evolutionary change on SR frequency in response to exposure to a novel stressor. This limits the extent to which our findings can be used to predict long-term SR dynamics in the wild. Specifically, we predict that linkage disequilibrium will lead to a disparity in the adaptive response of SR and non-SR genotypes to pesticides; this may reduce SR frequencies over evolutionary timescales if pesticide is applied persistently. In addition, we conducted no molecular analysis and are therefore limited to the extent we can attribute our findings to a specific molecular mechanism.

Although the overall trends demonstrated by our model are robust, we appreciate that, across much of the parameter space, the SR frequency predictions of our model are substantially higher than frequencies observed in the field [2,10]. There are three noteworthy assumptions made by our model that may have led to this outcome. First, some studies suggest that the fitness of SR males can become reduced with increasing levels of polyandry; this is because sperm competition disproportionately affects SR males due to their reduced ejaculate volume [16]. Our model assumes no impact of drive on a male’s ability to provide females with excess sperm and so ignores this phenomenon. Similarly, empirical evidence suggests that fitness in drive males is hindered at higher female densities due to an increased likelihood that drive males will become sperm depleted relative to non-drive males in response to repeated matings [25]. Although males in our model can mate multiple times, a male’s ability to supply a female with excess sperm is not impacted by his number of prior matings. Finally, there is evidence in stalk-eyed flies that drive alleles negatively affect sexually selected traits [48,49]. Our model does not take this into account. Had we included polyandry, negative impacts on sexually selected traits, and sperm depletion in our model, it is very likely that SR would have incurred a greater reproductive cost in males. Furthermore, this may have led to a reduction in model-predicted SR frequencies, such that our model output better mapped onto observed SR frequencies in wild populations. Nevertheless, the purpose of our model was to accommodate a general understanding of how the trends uncovered by our empirical data impact SR frequency, not to provide precise predictions for any specific system.

Our model parametrization is based on laboratory data. Although this is a common approach in many areas of theoretical biology, it can be considered a limitation due to the inherent exclusion of certain natural processes, which may bias what we observe/measure in a laboratory setting.

## 5. Conclusions

Meiotic drive is geographically widespread and can have profound effects on individual fitness and population sex ratio. However, we are limited in our ability to explain why, in the wild, drive does not always reach fixation and why there is such variation in drive frequency. Here, we show that drive has a considerable impact on female fecundity and increases mortality rate relative to non-drive individuals, both in the presence and absence of a novel environmental stressor. By extrapolating these findings using a mathematical model, we show that SR-frequency at the population level is highly sensitive to the intensity of a novel environmental stressor and variation in genotype-specific female fecundity. Wild populations frequently encounter novel stressors, and relative female fecundity varies both within and across species exhibiting drive. Thus, we believe these findings greatly enhance our ability to explain variation in drive frequencies across wild populations. Finally, given that our novel stressor of choice was pesticide, our work highlights the importance of acknowledging current pesticide use when considering the use of gene drives for pest control. Promising directions for future work could include an exploration of how additional environmental stressors, such as extreme temperatures and drought, affect drive frequency. In addition, studies exposing the molecular mechanisms behind the fitness impacts of drive would be particularly helpful in aiding our ability to understand and predict current and future drive frequencies.

## Data Availability

Raw data and analysis code is available in the supplementary material [50].
